# Uterine rupture in a primigravid patient, an uncommon but severe obstetrical event: a case report

**DOI:** 10.1186/s13256-017-1507-9

**Published:** 2017-12-06

**Authors:** Lotte Posthumus, Marielle Eveline Donker

**Affiliations:** 1 Spaarne Gasthuis, Spaarnepoort 1, 2134 TM Hoofddorp, The Netherlands; 2 Academisch Medisch Centrum, Meibergdreef 9, 1105 AZ Amsterdam, The Netherlands

**Keywords:** Uterine rupture, Primigravid, Unscarred uterus, Acute abdomen

## Abstract

**Background:**

A spontaneous rupture of the unscarred uterus in a primigravid patient is extremely rare and is associated with high perinatal and maternal morbidity and mortality.

**Case presentation:**

A 34-year-old white primigravid woman, 31 + 3 weeks of gestation, presented with pre-eclampsia and developed a sudden acute abdomen. An emergency laparotomy was performed and a uterine rupture was found as the cause of the event. A stillborn girl was born.

**Conclusion:**

A rupture of the pregnant uterus should always be considered in a pregnant woman presenting with abdominal pain, even in a primigravid patient.

## Background

Rupture of the pregnant uterus is an uncommon but severe obstetrical event, which is associated with high perinatal and maternal morbidity and mortality [1, 2]. It occurs mostly secondary to a previous caesarean section (the “scarred uterus”), making this its main risk factor [1, 2] with an incidence of around 1% [1]. The estimated incidence of a rupture of the unscarred uterus is 1/8000 to 15,000 deliveries [3, 4] or, as investigated by the World Health Organization (WHO), 0.006% [1]. Rupture in a primigravid patient is extremely rare and in most cases totally unexpected. The incidence of uterus rupture in general (primigravid or multigravid) is significantly higher in developing countries than in developed countries [1] caused by worse antenatal and obstetric care (such as periodic ultrasounds detecting for abnormalities and in-time cesarean section in case of prolonged or obstructed labor) [5], high frequency of home deliveries with prolonged labor, and grand multiparity [1, 5]. Until now there have only been 11 cases of a spontaneous rupture, without any risk factors [6–16], described in the English literature. Five of these cases were primigravid patients.

Here we report another case of a spontaneous rupture of an unscarred uterus in a primigravid patient, without any previous risk factors, at 31 + 3 weeks of gestation.

## Case presentation

A healthy 34-year-old white primigravid woman presented at 28 + 4 weeks of gestation with mild pre-eclampsia. She had no significant past medical history and her antenatal care had been uneventful. The pre-eclampsia was treated with intravenously administered magnesium sulfate (at admission) and methyldopa 750 mg 3 times daily and she received corticosteroids for accelerating fetal lung maturation. An ultrasound showed an intrauterine growth-restricted child in head position with an estimated fetal weight of 1047 gr and oligohydramnios. During admission, our patient was clinically and biochemically stable and daily cardiotocograms showed a reassuring fetal heart rate pattern. Two weeks after admission (30 + 4 weeks), the estimated weight of the fetus was 1116 gr with normal umbilical artery Doppler screening.

**Fig. 1:**
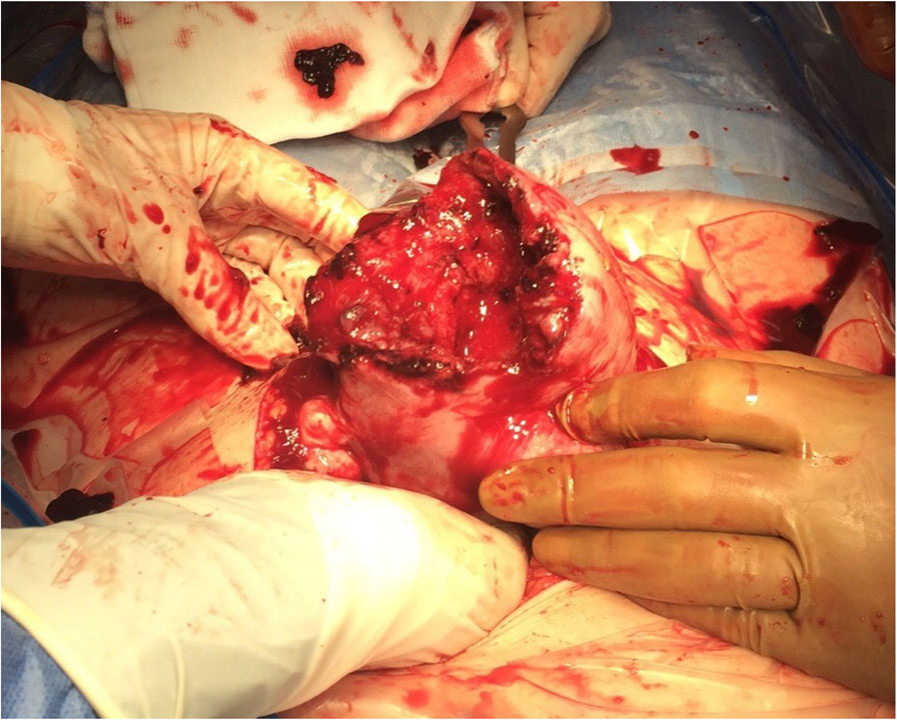
Rupture in the fundus of the uterus

At gestational age of 31+ 3 weeks, almost 2 weeks after admission, our patient complained about sudden lower abdominal pain and fever. There were no previous signs of preterm labor before this acute presentation. On clinical examination she looked pale with a blood pressure of 145/75 mmHg, a pulse of 103 beats per minute (bpm), a temperature of 37.9 °C, and a normal respiratory rate. On first physical examination her abdomen was soft but with slight tenderness in the lower abdomen. Ultrasonic evaluation showed an unviable fetus with no obvious signs of an abruption of the placenta. A vaginal examination revealed a closed portio and no vaginal bleeding. During the evaluation, she deteriorated with a blood pressure of 63/33 mmHg and a pulse of 130 bpm. She complained about an increased fluctuating abdominal pain and shoulder pain and showed difficulty in breathing. Her hypotension was considered due to intrauterine blood loss. Despite adequate fluid resuscitation, she remained hemodynamically unstable. She developed an acute abdomen. Ultrasound was repeated and showed free abdominal fluid. An emergency median laparotomy was performed and a hemoperitoneum of approximately 3 liters of blood was recovered. Both placenta and fetus were found outside the uterus due to a uterus rupture (Fig. 1). The tear was 5 cm long and located *in fundo* close to the insertion of the left tube. A stillborn girl with a weight of 1130 grams was born. Our patient’s uterus was closed in two layers. Blood and clots were removed. Our patient’s pelvis showed no abnormalities, especially no evidence of endometriosis or adhesions. Inspection of her liver showed no rupture. The placenta was sent for pathological examination. Syntocinon (oxytocin) was administered intravenously. There was an estimated total blood loss of 3500 cc. Six units of blood and 2 units of blood plasma were transfused.

In the days after surgery she developed an ileus, which was treated conservatively and she developed high fever with increased infectious parameters, due to small abscesses dorsal of her uterus, treated with antibiotics. A computed tomography (CT) scan showed a subcapsular liver hematoma without a decrease in her hemoglobin level or platelets level, which was treated conservatively. Blood cultures showed a *Staphylococcus aureus* infection. Endocarditis was excluded. An electrocardiogram (ECG) showed an intermittent second-grade atrioventricular (AV) block-type Wenckebach, without clinical consequence. The *S. aureus* infection was most likely a cause of an infected wound, which was treated with intravenously administered antibiotics, with a good response. One month after the event she was sent home. She was strongly advised not to get pregnant again. In the case of a new pregnancy, careful monitoring and an elective cesarean section were advised.

## Discussion and conclusions

Rupture of the non-laboring uterus is rare and can be life-threatening for the mother and fetus. Two reports [17, 18] on primigravid uterine rupture together show no uterine rupture among 52,876 primigravid deliveries over 10 and 13 years’ time. In developed countries the prevalence of uterine rupture in pregnant women with previous cesarean section has been reported to be approximately 1% whereas it is extremely rare in women without a history of cesarean section [1]. To the best of our knowledge, this is the sixth documented rupture of a primigravid uterus occurring before onset of labor without previous risk factors. All cases, except for one [12], were associated with symptoms of acute abdomen with prominent hemoperitoneum, occurring primarily in the third trimester. Four fetuses died before delivery, however, all patients recovered following adequate treatment [6–16]. The most common rupture sites were the cornual area and the uterine fundus. In our case, review of the first trimester ultrasound scans was unhelpful to assess the location of pregnancy. During surgery the tear in our patient’s uterus was close to the insertion of the left tube. This could have indicated a cornual or angular pregnancy, but first trimester ultrasound scans showed a normal location of pregnancy and the gestational growth was towards the uterine cavity. Furthermore, there was no (painful) asymmetrical enlargement of her uterus or abdomen with physical examination. Therefore, a cornual or angular pregnancy is considered not to be likely in this case.

The pathogenesis of rupture of the unscarred uterus is not well known. Associated factors are trauma (for example, domestic violence, traffic accident) and obstetric maneuvers (for example, internal version, breech extraction) which were not presented in this case. Medical induction or augmentation (oxytocin stimulation) of labor causes iatrogenic risk for rupture of the uterus [2, 19]. Other factors include other intrauterine surgeries, uterine anomalies [20–22], grand multiparity [2, 19, 23], abnormal placentation [22, 24], macrosomia [2], *in utero* exposure to diethylstilbestrol [25], cocaine abuse [26, 27], prolonged labor [2], and obstructed labor [7]. However, none of these factors were found in our patient. Ehlers–Danlos type IV has also been related to uterine rupture [28–30], but was excluded in our patient by genetic testing. In a review by Uccella *et al*. [31], 24 cases of prelabor uterine rupture in a primigravida were analyzed over the previous 60 years. Of 23 cases in which specific clinical data were available, 16 cases had a history of previous uterine surgery or instrumentation. Other identified risk factors included the ones mentioned above. Only one case had no clear risk factor [31].

During pregnancy, physiologic and anatomic changes may affect the presentation of abdominal pain [32–36]. Symptoms of normal pregnancy (nausea, vomiting, abdominal pain) could mimic severe abdominal pathology and fever is not always present [33–36]. Therefore, an acute abdomen during pregnancy often presents atypically and it could be difficult to distinguish a tense abdomen from a normal pregnancy [32, 37] and to detect an acute abdomen based on clinical and physical examination only [38].

Because of the rare incidence and the aspecific presentation, rupture of the uterus in our case was not the primary diagnosis. In combination with the pre-eclampsia, abdominal pain, tender lower abdomen, and negative heartbeat of the fetus, we thought of an abruptio placentae. It is unclear if the acute presentation was only due to the uterus rupture, or if this abruptio occurred prior to the rupture, making it a possible cause of the event [39]. Mourad *et al*. [14] recently also suggested that given the rarity of an idiopathic spontaneous uterine rupture in an unscarred primigravid uterus, a placental abruption is a more likely cause of a uterine rupture, especially in patients with pre-eclampsia [40]. In our case, pathological examination of the placenta showed signs of abruption and, therefore, in combination with the possible cornual or angular localization of the pregnancy, could be the cause of this severe obstetrical complication.

Rupture of the pregnant uterus is an uncommon but severe obstetrical event, which is associated with high perinatal and maternal morbidity and mortality [1, 2]. It often presents atypically, which makes it difficult to detect based on clinical and physical examination only. The main risk factor is a previous caesarean section (the “scarred uterus”), but a rupture of the pregnant uterus should always be considered in a pregnant woman presenting with abdominal pain, even in a primigravid patient.
